# SNHG 6 promotes the progression of Colon and Rectal adenocarcinoma via miR-101-3p and Wnt/β-catenin signaling pathway

**DOI:** 10.1186/s12876-019-1080-3

**Published:** 2019-09-18

**Authors:** Qianwen Shao, Jing Xu, Rong Deng, Wei Wei, Bing Zhou, Chao Yue, Miaoling Zhu, Haitao Zhu

**Affiliations:** 1 Department of oncology, The First Affiliated Hospital of Nanjing Medical University, Nanjing, Jiangsu People’s Republic of China 210029; 2 Genetic testing center, department of oncology, The First Affiliated Hospital of Nanjing Medical University, Nanjing, Jiangsu People’s Republic of China 210029; 3 General Department, Cancer hospital of NanJing Medical University, Nanjing, Jiangsu People’s Republic of China 210009; 4 Pathology department, Cancer hospital of NanJing Medical University, Nanjing, Jangsu People’s Republic of China 210009; 5 Colorectal cancer center, general surgery department, Jiangsu province cancer hospital, Affiliated Cancer hospital of NanJing Medical University, Jiangsu Cancer Institute, No 42, Baiziting road, NanJing, Nanjing, Jiangsu People’s Republic of China 210009

**Keywords:** Small nucleolar RNA host gene 6, ceRNA, Sponging, Target therapy, Cell signal pathway

## Abstract

**Background:**

Small nucleolar RNA host gene 6 (SNHG6) regulates diverse biological processes in cancers. Potential function of SNHG6 in human colon and rectal adenocarcinoma (CRC) was evaluated.

**Methods:**

Quantitative real-time polymerase chain reaction, MTT assays, Colony formation assays, Transwell assay, Western Blotting and Luciferase reporter assays were performed to measure the biological functions and potential molecular mechanisms of SNHG6 in CRC.

**Results:**

SNHG6 was over-expressed in CRC, and high expression of s SNHG6 were associated with short survival times. We then identified miR-101-3p as an inhibitory target of SNHG6. Knockdown of SNHG6 significantly decreased miR-101-3p expression. Moreover, silenced SNHG6 obviously inhibited CRC cell growth, weakened cell invasion capacity and blocked the Wnt/β-catenin signaling pathway.

**Conclusion:**

SNHG6 could regulate the progression of CRC via modulating the expression levels of miR-101-3p and the activity of Wnt/β-catenin signaling.

## Background

According to data of the American Cancer Society over the past decade, colon and rectal adenocarcinoma (CRC) remained the top three common cancer types, with 140, 250 estimated new cases and 50, 630 estimated deaths in 2018 [1]. Surgery seems to be the most effective therapeutic approach, but CRC patients still have about half experience recurrence [2]. Nowadays, cancer survival has improved due to treatment improvements, especially after adopting targeted therapies in CRC [3, 4]. Multiple non-coding RNAs have been proved to play important part in tumor development and progression [5–7].

Long non coding RNAs (lncRNAs) are a class of long non-coding RNAs, who exert significant functions as tumor suppressor or oncogene involving in tumorigenesis [8]. Accumulating evidences have consistently indicated that lncRNAs regulated tumorigenesis at both transcriptional and post-transcriptional level through sponging microRNA [9]. For instance, Yu F et al. reported that lincRNA-p21 suppressed human hepatic stellate cells activation via miR-17-5p-mediated-Wnt/β-catenin pathway [10]. Sun W et al. demonstrated that NEAT1_2 could function as a competing endogenous RNA to regulate AAA domain-containing protein 2 expression by sponging miR-106b-5p in papillary thyroid carcinoma [11]. Thus, the aberrant expression of lncRNAs could offer novel pathways of therapeutic targets for CRC.

In the present study, we revealed that SNHG6 was significantly up-regulated in human CRC tissues and cell lines. Over-expression SNHG6 was associated with shorter survival time. Moreover, we identified microRNA miR-101-3p as a negative regulation target for SNHG6. Silencing of SNHG6 suppressed CRC cells proliferation and invasion vitality. In addition, we uncovered that β-catenin and TCF4 were inhibitory targets of miR-101-3p, and that Wnt/β-catenin signaling was inhibited by miR-101-3p over-expression. Taken together, we demonstrated that the lncRNA SNHG6 promoted CRC progression via regulating the expression of miR-101-3p and the activity of the Wnt/β-catenin signaling pathway.

## Methods

### Clinical tissue samples

This research was conducted according to the World Medical Association Declaration of Helsinki and was approved by the ethics committee of Jiangsu province cancer hospital. Written informed consents were signed by all participants. Fifty five pairs of CRC tissues and adjust normal tissues were obtained from CRC patients in Jiangsu province cancer hospital from January, 2014 to March, 2016. Tumor tissues were diagnosed by pathological examination and all participants received no chemotherapy or radiotherapy before the surgery. Tumor tissues were stored at − 80 °C for later analysis.

### Cell culture and transfection

CRC cell lines HT29, SW620 and normal human intestinal epithelial cells HIECs were purchased from Shanghai Model Cell Bank (Shanghai, China). Cells were cultured in media Roswell Park Memorial Institute-1640 (RPMI-1640; Invitrogen, Carlsbad, CA, USA) or Dulbecco’s Modified Eagle’s Medium (DMEM; Gibco, Grand Island, NY, USA) supplemented with 10% fetal bovine serum (Gibco) at 37 °C in 5% CO_2_.

LncRNA SNHG6 siRNA, negative control (NC) siRNA, miR-101-3p and miR-101-3p mimics were all obtained from Gene-Pharma (Shanghai, China), and were transfected with Lipofectamine 2000 reagent (Invitrogen, Karlsruhe, Germany) according to the manufacturer’s instructions. Cells were seeded in 6-well plates at a concentration of 2 × 10^5^ cells/well, and were transfected with siRNA or miRNA when cells reached 40–60% confluence.

### Quantitative real-time polymerase chain reaction analysis (qRT-PCR)

At 48 h after transfection, total RNA was extracted from CRC tissues or cells using TRIzol reagent (Invitrogen) according to the manufacturer’s manual. Then, cDNA was generated by the PrimeScript RT Reagent Kit and qRT-PCR was performed using SYBR Premix Ex Taq (Takara Biotech, Dalian, China) according to the manufacturer’s manual. All qRT-PCR assays was performed on an ABI 7900 system (Applied Biosystems, Foster City, CA, USA). GAPDH or U6 was used as an internal control. Expression fold changes were calculated using 2^−ΔΔCt^ methods.

The primer sequences were as follows:

SNHG6, forward: 5′-CCTACTGACAACATCGACGTTGAAG-3′ and reverse: 5′-GGAGAAAACGCTTAGCCATACAG-3′;

GAPDH, forward: 5′-GGGAGCCAAAAGGGTCAT-3′ and reverse: 5′-GAGTCCTTCCACGATACCAA-3′.

MiR-101-3p, forward: 5′-UACAGUACUGUGAUAACUGA A-3′ and reverse: 5′-CAGUUAUCACAGUACUGUAU U-3′;

U6, forward: 5′-GCUUCGGCAGCACAUAUACUA AAAU-3′ and reverse: 5′-CGCUUCACGAAUUUGCGU GUCAU-3′.

### Luciferase assay

SNHG6-wild-type (SNHG6-Wt) was contracted by cloning the SNHG6 fragment containing the predicted miR-101-3p binding site into a pmirGlO Dual-luciferase miRNA Target Expression Vector (Promega, Madison, WI). SNHG6-mutated-type Vector (SNHG6-Mut) was contracted the same way but cloned SNHG6 fragment containing the mutated miR-101-3p binding site. Then vectors and miRNAs were co-transfected into 293 T cells at the indicated concentrations using Lipofectamine 2000 (Invitrogen) according to the instructions. After 48 h, luciferase activity was measured using a dual-light luminescent reporter gene assay kit (Promega).

### MTT assay

1 × 10^3^ CRC cells per well were seeded onto 96-well plate and incubated at 37 °C containing 5% CO2. Then, cells were washed twice with phosphate buffer saline (PBS; Thermo Fisher Scienti c, Waltham, MA) and 20 μL of methyl thi- azolyl tetrazolium (MTT; Thermo) solution was added to each well. 2 h later, cellular viability was detected by measuring the absorbance at 450 nm after 100 μL dimethyl sulfoxide (DMSO; Thermo) added to each well.

### Colony formation assay

0.5 × 10^3^ CRC cells per well transfected with indicated vector were seeded in six-well plates and cultured for two weeks. Colonies were then fixed in 10% formaldehyde for 10 min and stained with 0.5% crystal violet for 10 min. Finally, the number of visible colonies was counted manually.

### Transwell assay

1 × 10^5^ CRC cells transfected with indicated vector were seeded in the upper chambers of 8-μm pore size insert in the 24-well Transwell chamber (Costar, Boston, MA, USA) with 100 μl serum-free medium. The lower chambers were added with 500 μl medium containing 10% fetal bovine serum. After 48 h, the cells on the surface of the upper membrane were removed with a cotton tip, and the cells under the surface of the lower chamber were fixed with 4% paraformaldehyde for 20 min, stained with 0.1% crystal violet for 25 min. Invaded cells were counted in five randomly selected high-power fields. Experiments were performed in triplicate.

### Western blot analysis

Cells were lysed using RIPA protein extraction reagent (Beyotime, Shanghai, China). Then 25 μg protein extracts were separated by 10% sodium dodecyl sulfate polyacrylamide gel electrophoresis (SDS-PAGE), and then transferred onto nitrocellulose membranes (Millipore, Billerica, MA). Proteins were detected with specific primary antibodies against β-catenin, Cyclin D1, Axin2 or GAPDH (Santa Cruz, Dallas, Texas, USA) overnight. Horseradish peroxidase-linked secondary antibodies (Beyotime) were used as the second antibodies. Finally, protein blots were visualized with enhanced chemiluminescent substrate (Thermo).

### Statistical analysis

GraphPad prism software was used for statistical analysis, and data was presented as mean ± SD from at least three separate experiments. The significance of differences between groups were estimated by Student’s t-test, χ2 test or Wilcoxon test as appropriate. *P* < 0.05 was considered statistically significant.

## Results

### LncRNA SNHG6 were up-regulated in CRC

**Fig. 1:**
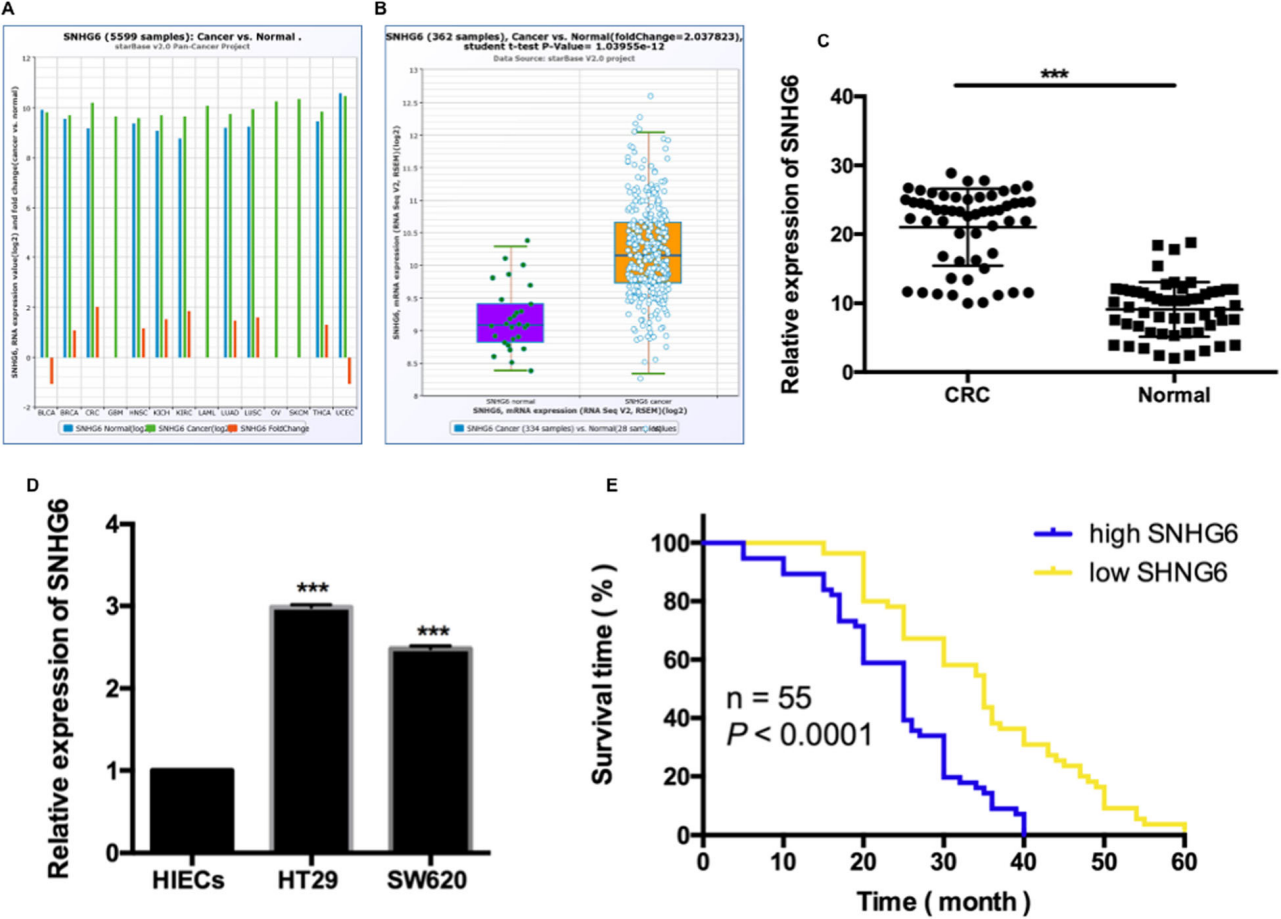
LncRNA SNHG6 were up-regulated in CRC. **a** The expression of SNHG6 among Pan-Cancer including 14 cancer types from The Cancer Genome Atlas (TCGA) Data Portal from starBASE v2.0. **b** The expression of SNHG6 in CRC or adjacent normal tissues from TCGA Data Portal. **c** The expression of SNHG6 was determined by qRT-PCR in 55 pairs of CRC tissues comparing with adjacent non-tumour tissues. **d** The expression of SNHG6 was examined by qRT-PCR in 2 CRC cell lines (HT29, SW620) and 1 normal human intestinal epithelial cell line HIECs. **e** High expression of SNHG6 was associated with shorter overall survival of CRC patients. *P < 0.05; **P < 0.01; ***P < 0.001

To explore the function of lncRNA SNHG6 in CRC development, we first investigated SNHG6 expression level in TCGA Data Portal from starBASE v2.0 [12, 13]. As Fig. 1a revealed, the expression of SNHG6 was over-expressed in 12 kinds of cancer tissues compared to the adjust normal tissues. Moreover, SNHG6 expression was significantly higher in CRC tissues compared to normal tissues (Fig. 1b; *P* < 0.001). To support this conclusion, we detected SNHG6 expression from 55 clinical CRC patient samples by qRT- PCR assays, which was ubiquitously increased compared to adjacent non-tumor tissues (Fig. 1c; *P* < 0.001). Next, SNHG6 expression was over-expressed in 2 CRC cells lines (HT29, SW620) comparing to human intestinal epithelial cells HIECs (Fig. 1d; P < 0.001). In addition, we performed Kaplan-Meier survival analysis to test the association between SNHG6 expression and the survival in 55 clinical CRC patients. High expression level of SNHG6 caused in much shorter survival time (Fig. 1e; *P* < 0.0001). Taken together, our results which was consistent with previous data suggested that SNHG6 might play a key role in CRC development and progression.

### miR-101-3p was an inhibitory target of lncRNA SNHG6

**Fig. 2:**
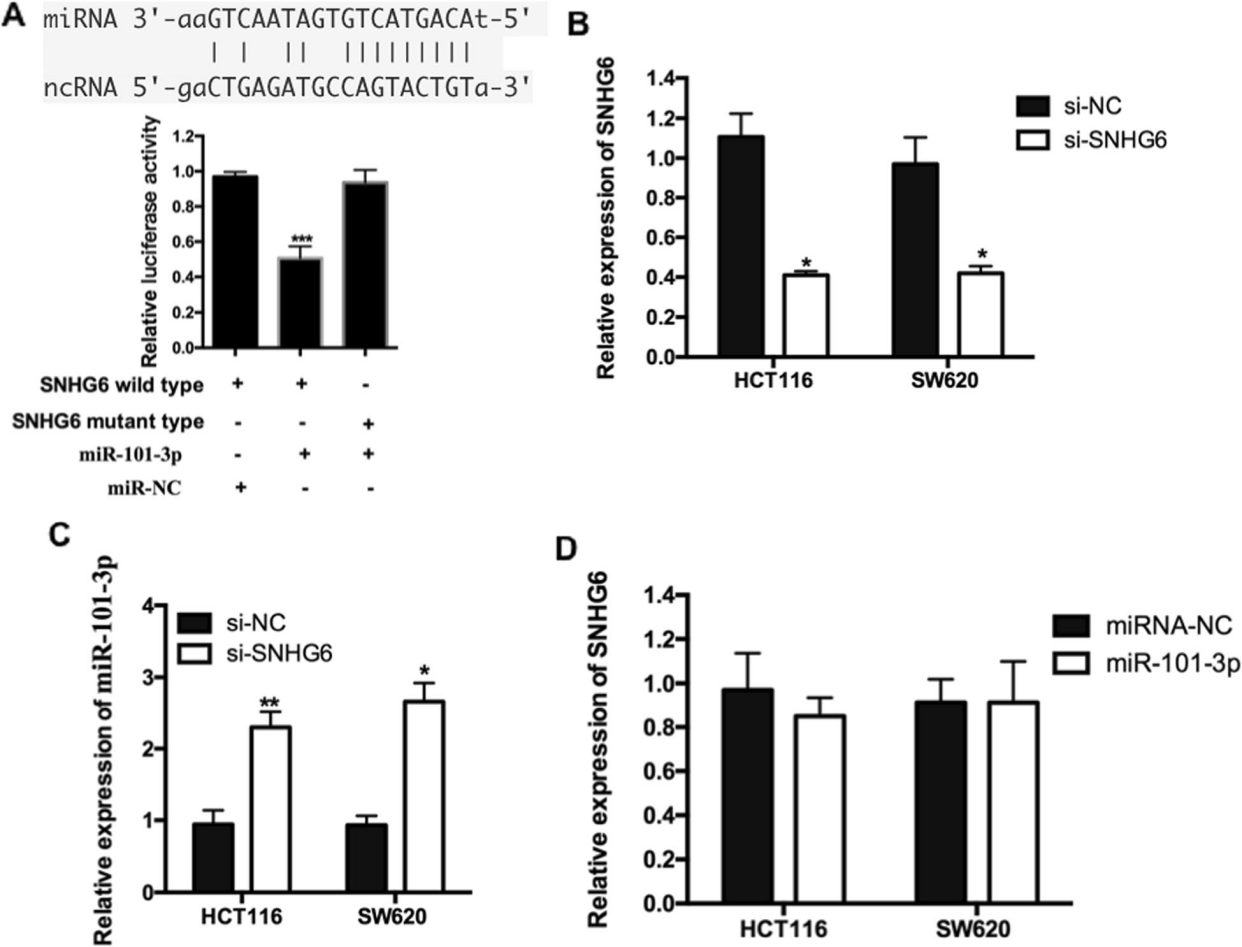
miR-101-3p was an inhibitory target of lncRNA SNHG6. **a** Sequence alignment of miR-101-3p with the putative binding sites with in the wild-type regions of SNHG6. Dual-luciferase reporter assay showed that miR-101-3p mimics reduced the intensity of fluorescence in HEK293T cells transfected with SNHG6-Wild type, while had no effect on the SNHG6-mutant vector. **b** Relative expression of SNHG6 after CRC cells transfected with si-SNHG6 or si-NC. **c** qRT-PCR analysis of miR-101-3p expression in HCT116 and SW620 cells transfected with si-SNHG6 or si-NC. **d** qRT-PCR analysis of SNHG6 expression in HCT116 and SW620 cells transfected with miR-101-3p or miR-NC. *P < 0.05; **P < 0.01; ***P < 0.001

Since recent evidences showed that lncRNAs negatively regulated miRNAs expression and activity in cancers [14, 15], we doubted if SNHG6 performed the similar molecular mechanisms in CRC. Firstly, we identified miR-101-3p as the potential target of SNHG6 by TCGA Data Portal from starBASE v2.0. The predicted miRNA target sites of miR-101-3p were showed in Fig. 2a. Ddual-luciferase reporter assay was performed after co-transfected with indicated vector and miRNA into 293 T cells, respectively. As shown in Fig. 2a, miR-101-3p significantly reduced wild type-SNHG6 luciferase activity, while have no effect on mutant-SNHG6 activity, which indicated that miR-101-3p bine to transcript position of SNHG6. To confirm that target, we knocked down SNHG6 by siRNA transfection. qRT-PCR analysis proved the significant silencing effect in two independent CRC cell lines, HT29 and SW620 cells (Fig. 2b; *P* < 0.05). Moreover, SNHG6 silencing significantly inhibited the expression of miR-101-3p in both CRC cell lines (Fig. 2c; P < 0.05), but miR-101-3p knock-down didn’t cause any change in the expression of SNHG6 (Fig. 2d; *P* > 0.05). These results supported that SNHG6 expression might be suppressed by miR-101-3p, and miR-101-3p was an inhibitory target of SNHG6.

### LncRNA SNHG6 promoted CRC cell proliferation and migration

**Fig. 3:**
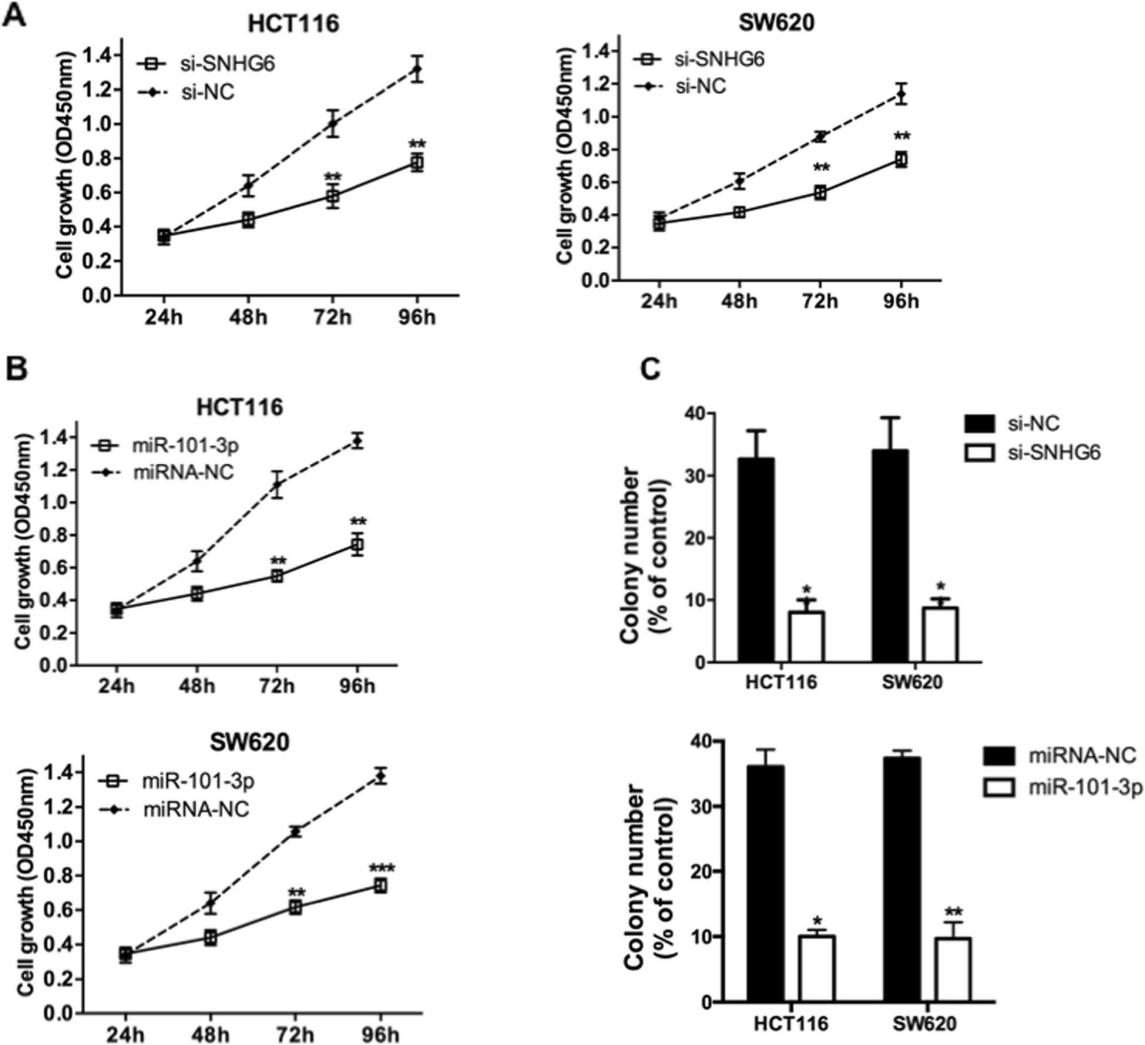
LncRNA SNHG6 promoted CRC cell proliferation and migration. **a** Effect of si-SNHG6 on cell proliferation of HCT116 and SW620 cells was detected by MTT assay. **b** Effect of si-SNHG6 on colony formation ability of HCT116 and SW620 cells was detected. **c** Effect of si-SNHG6 on cell invasion ability of HCT116 and SW620 cells was detected. *P < 0.05; **P < 0.01

To determine the function of SNHG6 on CRC cell proliferation, MTT assays were performed on two CRC cell lines that transfected with SNHG6-siRNA, respectively. SNHG6 inhibition significantly inhibited cell proliferation in both CRC cell lines compared to cells transfected with NC-siRNA (Fig. 3a; *P* < 0.05). We also performed additional assays to confirm the regulation of cell proliferation by SNHG6 and miR-101-3p. Colony formation ability of CRC cells transfected with SNHG6-siRNA were obviously suppressed comparing with cells in NC-siRNA group (Fig. 3b; P < 0.05). Next, invasion analysis was performed on CRC cells, and we found that CRC cells in SNHG6-siRNA group performed weaker invasion ability compared to NC-siRNA group cells (Fig. 3c; *P* < 0.05). These data collectively indicated that SNHG6 promoted CRC cell proliferation.

### miR-101-3p inhibited Wnt/β-catenin signaling by targeting β-catenin

**Fig. 4:**
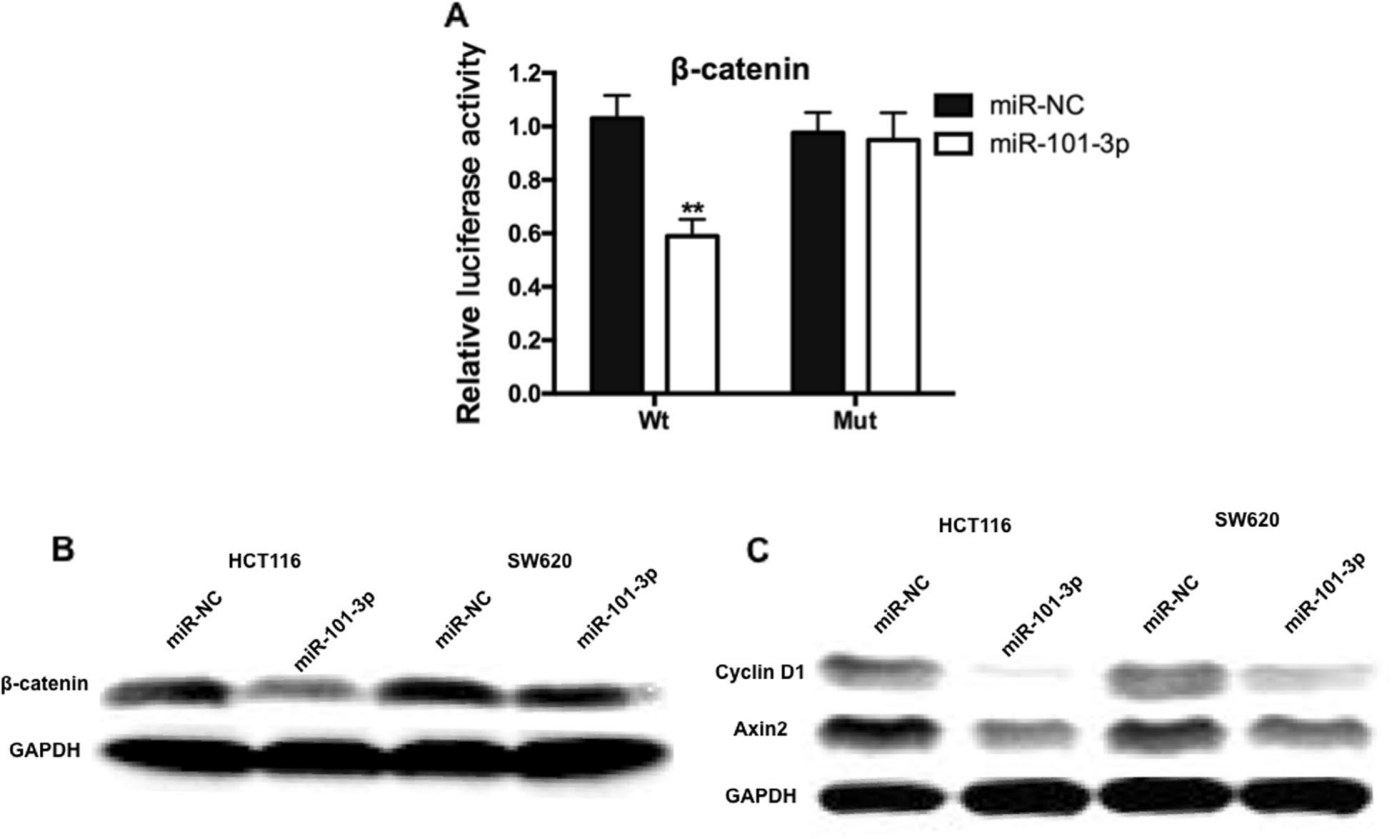
miR-101-3p inhibited Wnt/β-catenin signaling by targeting β-catenin and c-Myc. **a** Dual-luciferase reporter assay revealed that miR-101-3p inhibited wt β-catenin 3′-UTR luciferase activity, while it had no effect on Mut β-catenin 3′-UTR luciferase activity in HEK293T cells. **b** Expression of β-catenin and c-Myc were determined by western blot in HCT116 and SW620 cells transfected with miR-101-3p mimics or si-SNHG6. **c** The cartoon of the mechanism underlying the SNHG6-miR-101-3p-β-catenin axis in human CRC. **P < 0.01

Recent studies showed that miR-101-3p played crucial roles in cancer cell proliferation, and β-catenin in the Wnt/β-catenin signaling pathway were down-regulated after miR-101-3p transfection [16]. To figure out the molecular mechanisms of miR-101-3p oncogenic functions, the luciferase activity of intracellular signal transducer β-catenin was determined by co-transfecting β-catenin-Wt or β-catenin-Mut with miR-101-3p. We noticed that the luciferase activity of β-catenin was reduced in β-catenin-Wt group cells (Fig. 4a; *P* < 0.01). Furthermore, we discovered that the levels of downstream target genes of the Wnt/β-catenin signaling pathway, including β-catenin and c-Myc, were significantly suppressed by miR-101-3p over-expression or SNHG6 knockdown in CRC cells (Fig. 4b). Collectively, our data strongly support the hypothesis that SNHG6 promotes CRC progression and development via miR-101-3p mediated regulation of Wnt/β- catenin signaling (Fig. 4c).

## Discussion

Small nucleolar RNA host gene 6 (SNHG6), a newfound lncRNA located at chromosome 8q13.1, has been demonstrated to be a potential oncogene involved in the initial and development of various cancers, such as breast cancer [17], gastric cancer [18], hepatocellular carcinoma [19], and colorectal adenocarcinoma [20]. Yet, the biological roles and underlying mechanism of SNHG6 in CRC are largely unknown.

Nowadays, lncRNA including SNHG6 has been found to be a sponge for microRNA in multiply tumors. For example, in 2016, Chang L et al. suggested that SNHG6 promoted hepatocellular carcinoma growth and metastasis by endogenous competing miR-101-3p [21]. Then in 2017, Yan K et al. reported that SNHG6 could act as an oncogene in gastric cancer through competitively sponging miR-101-3p and silencing p27 [18]. The mature miRNA microRNA-101-3p (miR-101-3p) has been reported to been associated with carcinogenesis and cancer therapy in several malignancies [22, 23]. In this research, we investigated whether SNHG6 regulate the development and progression of CRC in a similar way. In our investigation, we found that SNHG6 expression was significantly increased in human CRC tissues and cell lines comparing to adjacent normal tissues or normal cells, and it was also corroborated by the analysis of available data in the TCGA database, which investing SNHG6 might play a great role in the progression of CRC. Moreover, we identified miR-101-3p as an inhibitory target of SNHG6 by luciferase reporter assay. Silencing of SNHG6 increased miR-101-3p expression, suggesting that miR-101-3p was the downstream of SNHG6. Furthermore, SNHG6 knockdown markedly inhibited CRC cells proliferation and invasion in vitro. Collectively, it documented that SNHG6 promoted carcinogenesis by acting as a miR-101-3p sponge in CRC.

Wnt/β-catenin signaling is known to regulate a broad range of cellular processes via regulating the expression of the multifunctional β-catenin protein, which is a crucial growth stimulatory factor in the Wnt/β-catenin pathway, leading to cell proliferation, invasion, differentiation and other signaling pathways [24, 25]. Mutated Wnt/β-catenin pathway components are causative to multiple growth-related pathologies and to cancer. Recent studies showed a significant correlation between intracellular signal transducer β-catenin in Wnt/β-catenin signaling pathway and miR-101-3p in tumor progression. For example, SNHG1 have been found to act as an oncogenic lncRNA promoting NSCLC tumorigenesis and progression via miR-101-3p/SOX9/ Wnt/β-catenin axis [26]. We then sought to confirm this prediction in the context of CRC cells. With dual-luciferase activity assay and western blot assay, the activity of Wnt/β-catenin signaling and the protein level of β-catenin and c-Myc (the downstream target genes of the Wnt/β-catenin signaling pathway) were substantially decreased by miR-101-3p over-expression or SNHG6 knockdown in CRC cells.

## Conclusion

We identified that SNHG6 could act as an oncogenic lncRNA that promoted CRC tumorigenesis and progression via miR-101-3p/Wnt/β-catenin axis, which providing a novel potential therapeutic target for the treatment of CRC.

## Data Availability

Not applicable.

## Abbreviations

CRC: Colon and rectal adenocarcinoma
qRT-PCR: Quantitative real-time polymerase chain reaction analysis
SNHG6: Small nucleolar RNA host gene 6
